# Case report: Novel variants in RELA associated with familial Behcet’s-like disease

**DOI:** 10.3389/fimmu.2023.1127085

**Published:** 2023-02-28

**Authors:** Jason W. An, Pallavi Pimpale-Chavan, Deborah L. Stone, Marcia Bandeira, Fatma Dedeoglu, Jeffrey Lo, John Bohnsack, Sofia Rosenzweig, Oskar Schnappauf, Dilan Dissanayake, Linda T. Hiraki, Daniel L. Kastner, Christina Pelajo, Ronald M. Laxer, Ivona Aksentijevich

**Affiliations:** ^1^ Division of Rheumatology, Department of Medicine, St. Michael’s Hospital, University of Toronto, Toronto, ON, Canada; ^2^ Division of Rheumatology, Department of Paediatrics, The Hospital for Sick Children, University of Toronto, Toronto, ON, Canada; ^3^ National Human Genome Research Institute (NHGRI), National Institutes of Health (NIH), Bethesda, MD, United States; ^4^ Division of Rheumatology, Hospital Pequeno Príncipe e Hospital de Clínicas, University Federal do Parana, Curitiba, Brazil; ^5^ Division of Immunology, Rheumatology Program, Department of Pediatrics, Boston Children’s Hospital, Harvard Medical School, Boston, MA, United States; ^6^ Department of Pediatrics, Spencer Fox Eccles School of Medicine, University of Utah, Salt Lake City, UT, United States

**Keywords:** RELA haploinsufficiency, Behcet’s Disease, RELA, NF-κB, RELA-associated inflammatory disease, RAID, mucosal ulcers, autoinflammatory

## Abstract

RELA haploinsufficiency is a recently described autoinflammatory condition presenting with intermittent fevers and mucocutaneous ulcerations. The RELA gene encodes the p65 protein, one of five NF-κB family transcription factors. As RELA is an essential regulator of mucosal homeostasis, haploinsufficiency leads to decreased NF-κB signaling which promotes TNF-driven mucosal apoptosis with impaired epithelial recovery. Thus far, only eight cases have been reported in the literature. Here, we report four families with three novel and one previously described pathogenic variant in RELA. These four families included 23 affected individuals for which genetic testing was available in 16. Almost half of these patients had been previously diagnosed with more common rheumatologic entities (such as Behcet’s Disease; BD) prior to the discovery of their pathogenic RELA variants. The most common clinical features were orogenital ulcers, rash, joint inflammation, and fever. The least common were conjunctivitis and recurrent infections. Clinical variability was remarkable even among familial cases, and incomplete penetrance was observed. Patients in our series were treated with a variety of medications, and benefit was observed with glucocorticoids, colchicine, and TNF inhibitors. Altogether, our work adds to the current literature and doubles the number of reported cases with RELA-Associated Inflammatory Disease (RAID). It reaffirms the central importance of the NF-κB pathway in immunity and inflammation, as well as the important regulatory role of RELA in mucosal homeostasis. RELA associated inflammatory disease should be considered in all patients with BD, particularly those with early onset and/or with a strong family history.

## Introduction

Behcet’s Disease (BD) is a rare, chronic inflammatory condition with mucocutaneous ulcerations, skin, ocular, joint, and vascular involvement. Its etiology is believed to be a combination of environmental factors in the background of multiple susceptibility genetic loci contributing to inflammation and immune dysregulation. The advent of whole-exome sequencing (WES) has identified monogenic causes of BD such as Haploinsufficiency of A20 (HA20), and more recently pathogenic heterozygous variants in the RELA Proto-Oncogene, NF-κB Subunit *(RELA)* gene (1–5).

*RELA* encodes the p65 protein, one of the five transcription factors belonging to the NF-κB family. The NF-κB pathway is critical in the regulation of the immune response, and dictates immune cell proliferation, apoptosis, and inflammation (6). Decreased NF-κB signaling promotes TNF-driven mucosal apoptosis with impaired epithelial recovery, whereas increased NF-κB signaling drives the generation of proinflammatory cytokines (7). Monoallelic pathogenic variants of *RELA* either cause a 50% dosage reduction (2) or generate a truncated protein with impaired function (4) resulting in RELA-associated autoinflammatory disease, that we have termed (RAID).

Three families have been reported to date in the literature. Badran et al. described a 3-year old girl with mucosal ulcerations, intermittent fevers, abdominal pain, and vomiting (2). Her mother had recurrent aphthous ulcers unresponsive to colchicine, and both were heterozygous for a pathogenic variant in the canonical splice site of *RELA* (c.559+1G>A; p.Thr164Profs*12). A second family described by Adeeb et al., reported five affected individuals across three generations, all of whom had mucosal ulceration and polymorphic rashes (4). A heterozygous mutation in *RELA*, c.1459delC; p.His487Thrfs*7, was shared by all affected patients. Although mucocutaneous ulceration seems to be the common clinical denominator, the spectrum of RAID may be broader. Comrie et al. reported a 5-year-old boy with a multisystem inflammatory disorder resembling autoimmune lymphoproliferative syndrome (ALPS) with cytopenias, splenomegaly, and slightly increased double negative T-cells (3). Despite the clinical phenotype, FAS-mediated apoptosis was normal. The proband was found to harbor a *de novo* heterozygous nonsense mutation in *RELA* (c.736C>T; p.Arg246*) (4).

Here, we report four additional families referred to the National Institutes of Health (NIH), Bethesda, USA, for clinical and genetic evaluation. Within these four families we identified four loss-of-function mutations in *RELA* in 15 patients (**Supplementary Table 1**). These mutations were predicted to be pathogenic based on the ACMG criteria and in silico analyses, but they are not functionally validated. All individuals consented to be reported anonymously and Research Ethics Board approval was obtained. Our case series adds to the overall understanding of the phenotypic and genotypic spectrum of RAID. It reaffirms the importance of attaining a molecular diagnosis in patients with early-onset and familial inflammatory disease given the implications in family planning and therapeutic choices discussed below.

## Case description

**Figure 1:**
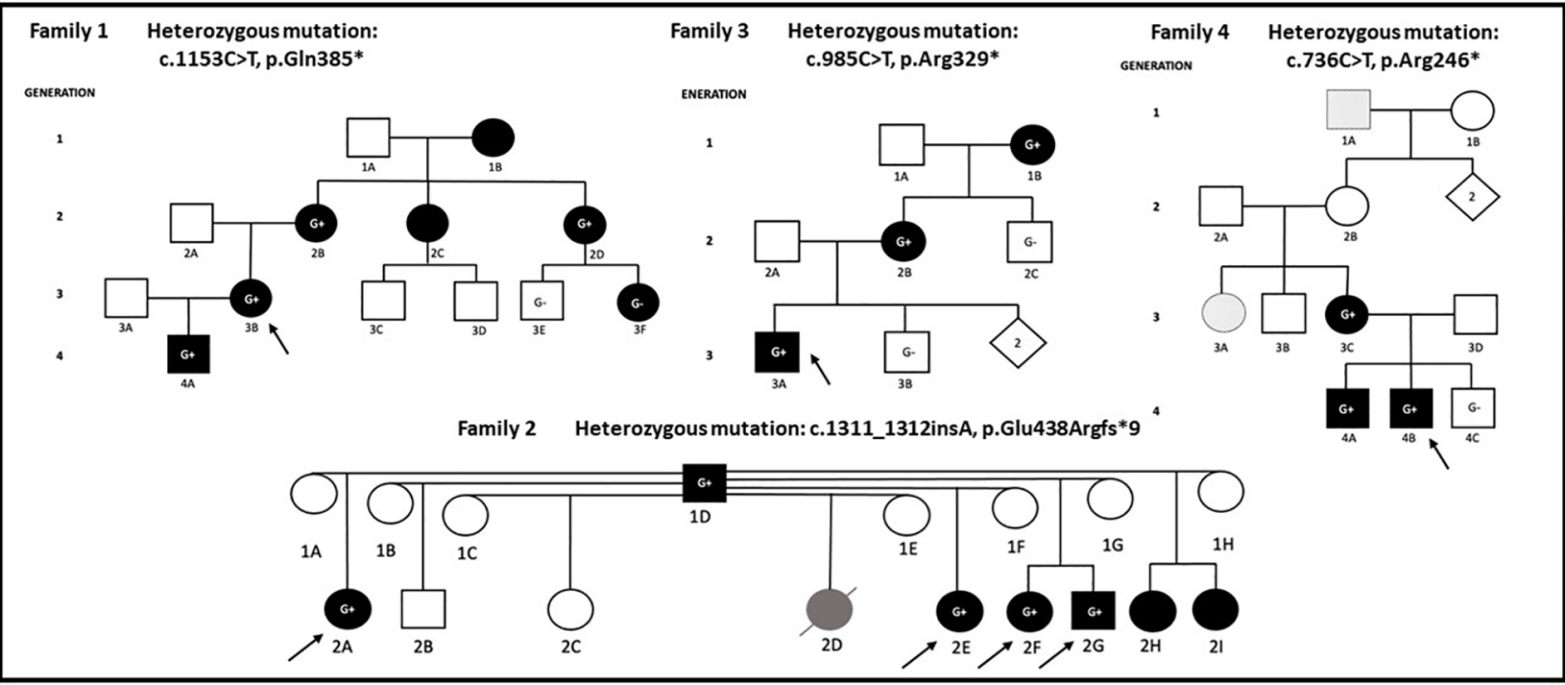
Proband in each family is denoted with arrow. Individuals shaded in black carry a combination of clinical features including mucocutaneous, musculoskeletal, gastrointestinal, neurological manifestations, infections, or fever. G+ denotes individual carrying pathogenic/likely pathogenic *RELA* variant, G- denotes wild type *RELA, i*ndividuals without ‘G’ were not sequenced. Patient (2D) in Family 2 died of lupus nephritis and is shaded in gray. Family 4: patients 1A and 3A presented with an isolated vesicular skin lesions and are shaded in dots.

We describe a four generation Canadian family (Family 1) of European ancestry with 7 individuals affected by mucocutaneous ulceration and autoinflammation (**Figure 1**).

**Figure 2:**
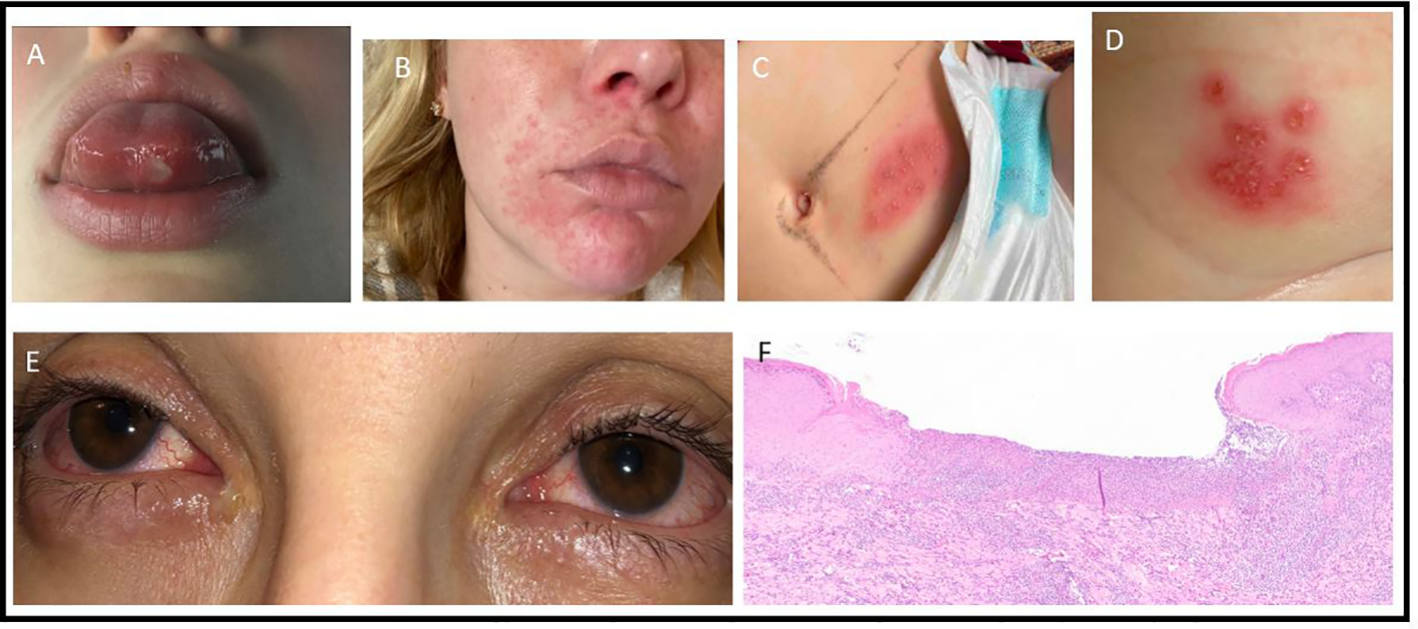
Clinical images. **(A)**: ulcer on tongue, F1-3B. **(B)**: facial rash, F1-3B. **(C)**: vesicular rash on lower abdomen, HSV negative, F4-4B. **(D)**: vesicular rash on inner thigh, F4-4B. **(E)**: conjunctivitis, F3-2B. **(F)**: Skin biopsy of F2-2G, showing an ulcerated lesion covered by fibrinoleukocytic exudate. The surrounding epidermis demonstrates discrete irregular epithelial hyperplasia. The dermis presents moderate lymphoplasmacytic and histiocytic infiltrate associated with neutrophilic exudates.

The proband (3B) is a 29-year-old female who suffered from monthly oral ulcers since early childhood and had one episode of vaginal ulceration at the age of 20. At the age of 25, she developed recurrent episodes of facial rash, arthralgias, myalgias, and night sweats occurring every 2 to 3 months and lasting 2 to 3 weeks (**Figures 2A, B**). Measured temperatures never exceeded 38C degrees. Her physical examination was unremarkable. Investigations revealed elevated c-reactive protein (CRP) ranging from 57-231mg/L during flares. Her complete blood count (CBC), creatinine, urinalysis, antinuclear antibodies (ANA), and liver enzymes were normal or negative. Infectious workups were consistently negative. She was diagnosed with BD and started methotrexate 20 mg subcutaneously weekly with folic acid. Despite this, she continued to have flares of severe oral ulcerations leading to multiple hospitalizations requiring intravenous corticosteroids. These flares prompted a referral to the Autoinflammatory Rheumatology Clinic in Toronto, Canada. Methotrexate was replaced with a trial of colchicine and etanercept, which resolved her oral ulcers. A commercial targeted gene sequencing panel revealed a heterozygous variant in *NLRP3* (p.Thr954Met). While it was initially classified as a Variant of Uncertain Significance, it has since been reclassified as Likely Benign in the ClinVar database (allele frequency =0.001; ACMG classification: BS1, BS2, BP6) (8). Furthermore, the *NLRP3* mutation (associated with Cryopyrin Associated Periodic Fever Syndrome) was inconsistent with the patient’s phenotype and no further segregation was performed. She was subsequently enrolled into a research study at the NIH, where additional gene panel testing (**Supplementary Material**) revealed a novel heterozygous variant in *RELA*, c.1153C>T, p.Gln385*.

On a closer review of her family history, it was determined that several other family members across four generations (1B/2B/2C/2D/3F/4A) had similar symptoms. The same pathogenic *RELA* variant was shared among the family members 4A, 2B, and 2D. Genetic testing results were unavailable for affected relatives 1B and 2C. The probands’ son (4A) was a previously healthy 9-year-old who developed monthly occurrences of painful oral ulcers on his lips, gums, and buccal mucosa. Physical exam was otherwise unremarkable. Bloodwork showed normal CBC, creatinine, liver enzymes, CRP, erythrocyte sedimentation rate (ESR), ANA, and immunoglobulin G/A/M. He was started on colchicine 1.2 mg daily with reduced occurrence of oral ulcers. The proband’s mother (2B) also had childhood-onset oral and genital ulcers. She was later diagnosed with neuro-Behcet’s based on severe headaches, fever, and brain fog. Her treatments have included prednisone, colchicine, and hydroxychloroquine. Her symptoms eventually subsided with menopause. The proband’s maternal aunt (2D) has had continuous oral ulcers since her mid-teens and was dependent on oral lidocaine. This has improved with age and colchicine. She has also had around 10 episodes of vaginal ulcers that seem to be triggered by stress. Body pains and fatigue were quickly aborted by short courses of prednisone. The proband’s cousin (3F) is a 26-year-old female who developed recurrent oral ulcers over the past 6 months at the time of writing. She does not have genital ulcers or fevers, but endorsed intermittent fatigue, myalgias, and polyarthralgias. Her primary rheumatologist believed that at least some of her complaints were associated with her depression and adjustment disorder. She has not required any medications thus far. On targeted gene sequencing, she tested negative for the *RELA* mutation. The proband’s maternal aunt (2C) had an onset of oral ulcers since the age of 8, and regular episodes lasted till she was 25. She had one genital ulcer around the age of 20. She did not have fevers or body pains and has never seen a rheumatologist. The proband’s maternal grandmother (1B) has suffered her entire life with oral ulcers, but no genital ulcers.

We describe a two generation Brazilian family (Family 2) where the father (1D) and 7 of his children 2A/2D/2E/2F/2G/2H/2I (mostly half-siblings of each other) are affected (**Figure 1**).

The father (1D) had a history of recurrent skin lesions labelled as allergies starting in early life. There was no history of any other systemic manifestations, and he is not on any treatment. DNA samples from four affected patients in the second generation were sent to NIH for genetic testing. Patient 2A is a female with recurrent oral and genital ulcers starting at 2 years of life. Over the years she developed fatigue, arthralgia, atopic dermatitis, periodontitis, repeated episodes of folliculitis, ulcerated lesions on the lower limbs, and headaches. Patient 2E is a female with recurrent oral ulcers starting at 3 years of life. She later developed fever, arthralgias, genital and oral ulcers, atopic dermatitis, folliculitis, erythematous papular lesions on the lower limbs, abdomen, and axilla with central ulcerations. A biopsy from the affected skin was suggestive of psoriasiform superficial dermatitis with perivascular inflammatory infiltrate but absent vasculitis. Patient 2F is a female who was born at term and admitted to the NICU for pneumonia, culture negative sepsis, and pneumothorax. At 40 days of life, she developed otitis media and cellulitis. At 6 months of life, she had bloody stools and at 8 months developed oral ulcers and fevers. She continued to have recurrent oral, peri-anal, and genital ulcers, otitis media, pharyngitis, and recurrent pustular rash, erythematous papular lesions over the buttocks, folliculitis, renal calculi, and appendicitis leading to intestinal necrosis requiring ileocolectomy. Following the surgery, she complained of recurrent abdominal pain and was diagnosed with esophagitis and chronic follicular colitis. At age 12, she had recurrent headaches and MRI brain showed multifocal white matter lesions compatible with hemoglobin degradation deposits. Her full brother, patient 2G, had disease onset at 5 years of age with recurrent oral, genital, and anal ulcers. He later developed folliculitis and ulcerative skin lesions for which biopsy showed chronic inflammation (**Figure 2F**). He also had recurrent headaches with a normal brain MRI. All four children were diagnosed with BD. While 2A, 2F, 2G had elevated acute phase reactants during some episodes, 2E did not. 2E and 2F were ANA positive while 2A and 2G were ANA negative. All four siblings received prednisolone, colchicine, and various immunosuppressive therapies (**Supplementary Table 2**). Positive responses were observed with infliximab and adalimumab. Half-siblings 2B and 2C did not report any symptoms to date. Another half-sibling, 2D, was diagnosed with systemic lupus erythematosus (SLE) and died at 6 years of age due to complications from renal lupus. Siblings 2H and 2I report history of oral ulcers, skin lesions, and recurrent scalp lesions respectively.

The four affected children (2A/E/F/G) were tested on the targeted gene panel at NIH and found to carry a novel heterozygous frameshift mutation (c.1311_1312insA, p.Glu438Argfs*9) in *RELA*. Sanger sequencing confirmed the same variant in the father. Due to non-availability of samples, genetic testing was not performed on siblings 2B, 2C, 2D, 2H, and 2I.

We describe a third, American family (Family 3) of mixed European ancestry with three affected individuals across three generations (**Figure 1**).

The proband (3A) is a 9-year-old boy with recurrent fevers, peritonitis, bloody diarrhea, and arthralgias. Endoscopy showed enlarged lymphoid aggregates in duodenal mucosa and mild cryptitis in the transverse colon. CT of his abdomen revealed mesenteric lymphadenitis. He had a normal/negative ANA, C3/C4, immunoglobulins, and vaccine responses. He had elevated ESR, CRP, fecal calprotectin, leukocytosis (neutrophilic predominance), and TNF levels. Immunophenotyping showed increased number of CD19^+^ B cells. Treatment with colchicine and anakinra were not effective, whereas etanercept resulted in improvement. His mother (2B) had recurrent fevers, knee swelling, and elevated ESR and CRP since the age of 10. She also had tendonitis, conjunctivitis, sicca, and a history of 2 miscarriages (**Figure 2E**). Skin involvement included a thigh rash with leukocytoclastic vasculitis on biopsy, as well as possible erythema nodosum. She was given previous diagnoses of Sjogren’s syndrome and SLE. The proband’s maternal grandmother (1B) also had abdominal symptoms starting at 10 years of age, leading to a colonoscopy that showed inflammation.

Genetic testing on a commercial targeted gene sequencing panel revealed a novel non-sense mutation (c.985C>T, p.Arg329*) in *RELA* shared between the proband, his mother, and maternal grandmother. The proband’s maternal uncle (2C) had nonspecific gastrointestinal symptoms and tested negative for the *RELA* mutation.

The fourth American family (Family 4) of mixed European ancestry includes five affected individuals across four generations (**Figure 1**).

The proband (4B) exhibited hypotonia and poor weight gain since birth. At 6 months, he developed febrile flares with oral ulcers and diarrhea. He also had muscle weakness, vesicular rash (**Figures 2C, D**) and skin ulcerations (in the abdomen, groin, thigh, peri-rectal areas). He was referred to the immunodeficiency clinic with a history of infections with respiratory syncytial virus, croup, and otitis media. Herpes was suspected, but vesicular cultures were negative, and he did not respond to empiric valacyclovir. ESR was elevated during a flare of his rash. Flow cytometry showed elevations in CD4^+^ T-cells, with predominance of naïve CD45RA subsets and elevated HLA-DR markers of activation. Prednisolone was given for 5 days at the time of flare, and he was later started on etanercept, both of which he responded well to. His older brother (4A) presented with non-specific abdominal pain. The mother (3C) had ocular inflammation, and the maternal aunt (3A) had vesicular cervical lesions. The maternal great-grandfather (1A) had facial lesions previously labelled as shingles. Empiric antiviral therapy was ineffective.

Genetic testing on in 3C, 4A, and 4B using a commercially designed targeted gene panel revealed a previously reported nonsense mutation (c.736C>T, p.Arg246*) in *RELA*. We were unable to obtain samples to genotype 3A, 3B, 2B, and 1A.

The clinical manifestations of all 15 *RELA* mutation-positive patients, their clinical diagnoses prior to genotyping, and therapies are summarized in **Supplementary Table 2**.

## Discussion

Our case series describes 15 patients from four families carrying three novel disease-associated mutations in *RELA*, adding to the eight patients currently published in the literature. The clinical heterogeneity was broad; the most common features were oral and genital ulcers (**Table 1**). The least common were conjunctivitis and recurrent infections.

**Table 1:** Prevalence of clinical features in our series of 15 RAID patients from 4 families.

Clinical Manifestation	Number of patients (n = 15)	%
Oral ulcers	9	60
Genital ulcers	8	53
Rash^1^	7	47
Carried prior alternative diagnosis^2^	7	47
Fevers (T>38C)	6	40
Arthralgias/ Arthritis	6	40
Gastrointestinal^3^	5	33
Headaches	4	27
Conjunctivitis	2	13
Recurrent infections	2	13

^1.^ Includes maculopapular, eczematoid, folliculitis, psoriasiform, ulcers, erythema nodosum, vesicular, leukocytoclastic vasculitis. Areas affected include truck, limbs, face, scalp.

^2.^ 47% of RAID patients carried other diagnoses prior to discovery of their pathogenic RELA variant. See **Supplementary Table 2** for further details.

^3.^ Gastrointestinal features in these 5 patients included recurrent abdominal pain, bloody stools, chronic diarrhea, appendicitis, intestinal necrosis, esophagitis, colonoscopy showing (inflammation, cryptitis, or follicular colitis).

^4.^ Infections include respiratory syncytial virus, croup, otitis media, pneumonia, cellulitis, pharyngitis. Patients with RAID had symptoms (above), in conjunction with a pathogenic variant in *RELA*.

**Figure 3:**
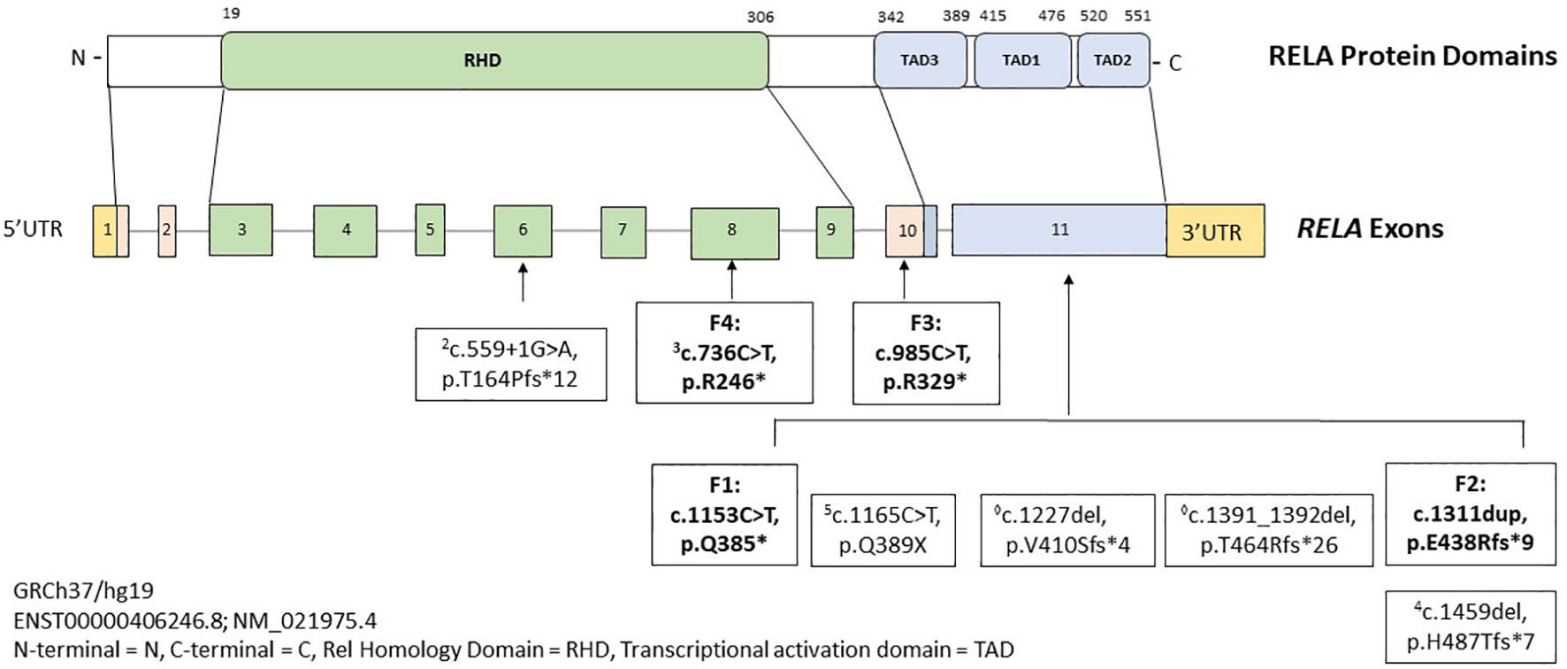
Schematic showing domain structure and functional motifs of the transcription factor p65 protein with summary of pathogenic/likely pathogenic variants in *RELA* gene. Mutations reported in literature are depicted in regular font and in bold are mutations identified in our four families. Superscripted numbers correspond to the original cases listed in References.

Pathogenic variants in *RELA* drive an autosomal dominant inflammatory syndrome marked by mucosal ulceration, fevers, arthritis, gastrointestinal inflammation, and rash, which we have named RAID. **Figure 3** depicts all RAID-associated mutations including those discovered in this study. In addition to BD-like symptoms, RAID can present with broader autoimmunity and mimic classic rheumatologic diseases and even infections (**Supplementary Table 2**). For example, features of SLE were observed in two cases in our cohort. In Family 1, patient 2B had high titer anti-dsDNA antibodies. In Family 3, patient 2B was clinical diagnosed with SLE. Interestingly, patient 2D in Family 2 was diagnosed with classic SLE and died from complications of lupus nephritis. As her DNA sample was unavailable, we could not confirm her *RELA* genotype or she developed severe SLE at an unusually young age. Two observations from the literature suggests that loss-of-function in *RELA* may promote SLE manifestations. First, a recent study (preprinted in BioRxiv) identified novel *RELA* mutations in 3 early-onset cases of SLE (9). The authors reported that the p65 mutants translocated to the nucleus but were transcriptionally inactive towards NF-κB genes. Instead, strong induction of IFN-stimulated genes were observed. Given the well-established type 1 IFN signature in SLE, the authors concluded that *RELA* haploinsufficiency could promote SLE development. Second, Haploinsufficiency of A20 (HA20) is another disorder which is driven by constitutive upregulation in the canonical NF-κB pathway (10). A20 is a ubiquitin-editing enzyme which negatively regulates pro-inflammatory signaling components (such as NEMO and RIPK1), of the NF-κB pathway (11). Some patients with HA20 produce low titer ANA and/or develop SLE features such as nephritis, CNS vasculitis, and intradermal mucin accumulation on skin biopsy (12). Other autoimmune phenotypes have also been associated with pathogenic *RELA* variants, such as a 22-year-old girl who developed neuromyelitis optica with anti-aquaporin 4 antibodies (4), and a 5-year-old boy presenting with ALPS (3). In a similar observation, patients with HA20 can also present with an ALPS-like phenotype (13). The *RELA* variant (p.Arg246*) associated with ALPS was the same as one found in our patient who presented with oral ulcers, fevers, diarrhea, and rash.

Given the integral role of both A20 and *RELA* in the NF-κB pathway, these findings suggest that dysregulation in cell-death pathways may affect both hematopoietic cells and epithelial cells (14, 15). While *RELA* pathogenic variants are sufficient to produce an inflammatory phenotype, the specific features are likely modified by genetic, epigenetic, and environmental factors (such as infection).

The features of BD exhibited in patients with RAID and HA20 highlights the concept of BD as a syndrome rather than a distinct disease entity. The etiology of BD appears to be multifactorial in most cases but may be monogenic in some. Some clinical features may be unusual for classic multifactorial BD and may alert the clinician to a possible monogenic disease (5). Examples may include pediatric onset, positive family history of inflammatory diseases, recurrent fevers, positive autoantibodies, or features of a seemingly unrelated concurrent autoimmune disease. We compared and contrasted features of monogenic and polygenic BD in **Supplementary Table 3**.

In this report, we observed that pathogenic variants in *RELA* exhibit variable expressivity. Patient F2-1D presented with nonspecific skin rashes, while 4/7 of his affected mutation-positive children had a multitude of severe early-onset symptoms. The pedigree in Family 4 also raises suspicion of incomplete penetrance in RAID, which has not been previously reported. Patient 1A had blistering skin lesions (unresponsive to antiherpetic therapy) while his daughter (2B) was asymptomatic. Both lacked genetic diagnoses. 2B has an affected daughter 3C, who presented with isolated conjunctivitis, and her own children 4A and 4B presenting with genital lesions and ocular inflammation. An alternative explanation is that the RELA pathogenic mutation occurred *de novo* in patient 3C, while 3A and 1A (neither genotyped) coincidently had blistering lesions.

Finally, the phenotypic spectrum of RAID may not be purely limited to inflammation. We report recurrent infections in 2/15 RAID patients (F4-4B, F2-2F), as may be expected due to impaired NF-κB signaling in leucocytes. Furthermore, a report from 2016 described a 7-day-old neonate who died of unknown causes and was found to have high bone mass on radiographs and histology (16). WES revealed a *de novo* heterozygous missense variant in *RELA* (c.1534_1535delinsAG, p.Asp512Ser). Based on the previous reports that abnormal NF-κB signaling results in increased osteoblast differentiation and activity, the authors speculated that the missense mutation might exert a dominant-negative effect on *RELA* and promote excessive bone formation. The functional consequences of this variant have not been validated, neither we observed bone abnormalities in our patients’ cohort.

Patients in our series were treated with a variety of medications including glucocorticoids, colchicine, methotrexate, TNF inhibitors, thalidomide, and anakinra (**Supplementary Table 2**). Benefit was observed with glucocorticoids, colchicine, and TNF inhibitors (etanercept specifically). Other studies have reported benefit with infliximab (2), etanercept (4), mycophenolate, intravenous immunoglobulin, steroids, and rituximab (3).

## Conclusions

The NF-κB pathway regulates inflammation and immune responses in hematopoietic, epithelial, and other cell types. Timing, signal strength, tissue type, exposure to infections, microbiome, genetic and epigenetic modifiers may influence the phenotypic variation observed in patients with pathogenic mutations in key NF-κB components, including *RELA* (7). Our results show that loss-of-function mutations in *RELA* are typically associated with familial BD-like disease, but a wide phenotypic spectrum can be seen with features of other classic rheumatologic diseases (**Supplementary Table 2**). Monogenic entities such as RAID or HA20 should be considered in patients with early-onset BD-like or other autoimmune syndromes especially in the context of a positive family history. These patients should undergo targeted genetic testing including both *RELA* and *A20*, if not exome sequencing. The current data do not support a correlation between the specific mutation and clinical manifestations, but further insight may be gained in a larger cohort of patients. Patients diagnosed with RAID may benefit from glucocorticoids, colchicine, and TNF inhibitors.

## Limitations

The main limitation of this study is the lack of functional validation for the new mutations reported here. The causality was interpreted based on their family segregation, absence in all public genetic databases, and in silico predictions for the impact of these loss-of-function variants on protein function.

## Strengths

Given the relatively new discovery of RAID, the addition of our 15 patients double the number of cases described in the literature and further highlights the broad clinical spectrum of RAID.

## Data availability statement

The original contributions presented in the study are included in the article/**Supplementary Materials**, further inquiries can be directed to the corresponding author.

## Ethics statement

The studies involving human participants were reviewed and approved by Institutional Review Board of the National Institutes of Health, Bethesda, USA. Written informed consent to participate in this study was provided by the participants’ legal guardian/next of kin. Written informed consent was obtained from the individual(s), and minor(s)’ legal guardian/next of kin, for the publication of any potentially identifiable images or data included in this article.

## Author contributions

All authors contributed to the article and approved the submitted version. JA and IA wrote the manuscript, tables and figures. PP-C contributed to figures. DLS, MB, FD, JL, JB, DD, DLK identified and contributed patients to this study. Specifically, OS, SR, and IA performed genetic testing, DD and LH contributed to genetic testing. RL, and IA provided overall direction and guidance to the work.
