# Supplementary material for: Case report: Novel variants in RELA associated with familial Behcet’s-like disease
Source: Front Immunol. 2023 Feb 28;14:1127085. doi: 10.3389/fimmu.2023.1127085 (PMC10011480; doi:10.3389/fimmu.2023.1127085)
Supplement: Supplementary file 1 [file Table_1.docx]

**Supplementary Table 1:** Bioinformatic features of four *RELA* variants found in our four families.

| **Family** | **Genomic coordinates** | **Codon, amino acid** | **Consequence** | **Mutation Taster** | **CADD** | **ACMG** |
| --- | --- | --- | --- | --- | --- | --- |
| 1 | chr11- 65422352 G>A | Heterozygous  c.1153C>T, p.Q385* | Stop gain | Disease causing | 33 | Pathogenic (PVS1, PM2, PP5) |
| 2 | chr11- 65422194dup | Heterozygous  c.1311dup, p. E438Rfs*9 | Frameshift | NA | NA | Likely Pathogenic (PVS1, PM2) |
| 3 | chr11- 65423207 G>A | Heterozygous  c.985C>T, p.R329* | Stop gain | Disease causing | 38 | Pathogenic (PVS1, PM2, PP5) |
| 4 | chr11- 65425899 G>A | Heterozygous  c.736C>T, p.R246* | Stop gain | Disease causing | 38 | Pathogenic (PVS1, PM2, PP5) |

**GRCh37/hg19; ENST00000406246.8; NM_021975.4**

**gnomAD pLI = 1; All four variants are not found in gnomAD genomes or exomes**

**Abbreviations:** Combined Annotation Dependent Depletion = CADD**,** American College of Medical Genetics and Genomics = ACMG

ACMG^10^criteria:

PVS1 (Pathogenic very strong) criteria: Null variant (stop gain/frameshift duplication) in a gene where loss of function is a known mechanism of disease, can be considered a very strong piece of evidence for pathogenicity.

PM2 (Pathogenic moderate) criteria: If a variant is absent from a large general population or a control cohort (>1000 individuals) and the population is race-matched to the patient harboring the identified variant, then this observation can be considered a moderate piece of evidence for pathogenicity.

PP5 (Pathogenic supporting) criteria: Reputable source recently reports variant as pathogenic, but the evidence is not available to the laboratory to perform an independent evaluation, can be considered a supporting piece of evidence for pathogenicity.
