# Supplementary material for: Case report: Novel variants in RELA associated with familial Behcet’s-like disease
Source: Front Immunol. 2023 Feb 28;14:1127085. doi: 10.3389/fimmu.2023.1127085 (PMC10011480; doi:10.3389/fimmu.2023.1127085)
Supplement: Supplementary file 2 [file Table_2.docx]

**Supplementary Table 2**: Details of clinical features in our cohort of RAID patients

| **Epidemiology** | | | **Genetics** | **Clinical Features** | | | | | | | | **Previous diagnostic labels (prior to genetic sequencing)** | **Treatment** |  |
| --- | --- | --- | --- | --- | --- | --- | --- | --- | --- | --- | --- | --- | --- | --- |
| **Family** | **Pt** | **Age of onset, Sex** | **Genotype** | **Fever** | **Oral Ulcers** | **Genital Ulcers** | **Rash** | **Joints** | **GI** | **Headache** | **Other** |  | **Tried** | **Effective** |
| **1** | 2B | 8, F | p.Gln385* |  |  |  |  |  |  |  |  | Neuro-Bechet | GC, COL, HCQ | Data Unavailable |
|  | 2D | 15, F | p.Gln385* |  |  |  |  |  |  |  |  |  | GC | GC |
|  | 3B | 29, F | p.Gln385* |  |  |  |  |  |  |  |  |  | MTX, GC, COL, ETN | GC, COL, ETN |
|  | 4A | 9, M | p.Gln385* |  |  |  |  |  |  |  |  |  | COL | COL |
| **2** | 1D | Unkn., M | p.Glu438Argfs*9 |  |  |  |  |  |  |  |  |  | None |  |
|  | 2A | 2, F | p.Glu438Argfs*9 |  |  |  |  |  |  |  | Periodontitis | Behcet’s Disease | GC, MTX, COL, THAL, INF, ADA | INF, ADA |
|  | 2E | 3,F | p.Glu438Argfs*9 |  |  |  |  |  |  |  |  | Behcet’s Disease | GC, COL, MTX, INF | INF |
|  | 2F | 40d, F | p.Glu438Argfs*9 |  |  |  |  |  |  |  | NICU for pneumonia, culture –ve sepsis, cellulitis, otitis media, pneumothorax. | Behcet’s Disease | GC, COL, MTX, ADA, AZA | ADA |
|  | 2G | 5, M | p.Glu438Argfs*9 |  |  |  |  |  |  |  |  | Behcet’s Disease | GC, COL, MTX, ADA, AZA | ADA |
| **3** | 1B | 10, F | p.Arg329* |  |  |  |  |  |  |  |  |  | Data Unavailable |  |
|  | 2B | 10, F | p.Arg329* |  |  |  |  |  |  |  | Sicca, conjunctivitis | Sjögren’s, SLE | GOL, ADA |  |
|  | 3A | 9, M | p.Arg329* |  |  |  |  |  |  |  |  |  | COL, ANA, ETN | ETN |
| **4** | 3C | 26, F | p.Arg246* |  |  |  |  |  |  |  | Conjunctivitis |  | Data Unavailable |  |
|  | 4A | 2, M | p.Arg246* |  |  |  |  |  |  |  |  |  | No treatment | Data Unavailable |
|  | 4B | Birth, M | p.Arg246* |  |  |  |  |  |  |  | Hypotonia, poor weight gain, RSV, croup, otitis media | Herpes rash (culture negative) | GC, ADA | GC, ADA |

**Supplementary Table 2**: Heat map with colored columns signifying positive clinical features in our cohort. All 15 individuals included above had various constellation of symptoms. Abbreviations: AZA = azathioprine. COL = colchicine. ETN = etanercept. GC = glucocorticoids. GOL = Golimumab. HCQ = hydroxychloroquine. INF = infliximab. MTX = methotrexate. RSV = Respiratory Syncytial Virus. SLE = systemic lupus erythematosus. THAL = thalidomide
