# Supplementary material for: Case report: Novel variants in RELA associated with familial Behcet’s-like disease
Source: Front Immunol. 2023 Feb 28;14:1127085. doi: 10.3389/fimmu.2023.1127085 (PMC10011480; doi:10.3389/fimmu.2023.1127085)
Supplement: Supplementary file 3 [file Table_3.docx]

**Supplementary Table 3 - Classic vs Monogenic Behcet’s Disease**

| **Clinical Features** | **Classic Polygenic BD** | **Monogenic BD** | |
| --- | --- | --- | --- |
|  |  | **RAID** | **HA20** |
| Onset | Early - mid adulthood | Pediatric | Pediatric |
| Family History | Usually -ve | +ve for ‘BD’ +/- other inflammatory syndromes | |
| Oral/ Genital Ulcers | + | + | + |
| GI | + | + | + |
| Skin | + | + | + |
| Musculoskeletal | + | + | + |
| Fevers | Unusual | Common | Common |
| Neurologic | + | + | + |
| Ocular | + | + | + |
| Pathergy | + | unknown | + |
| Genetic factors | Polygenic: enriched for HLA-B51 haplotype and other risk alleles | Monogenic: *RELA* pathogenic variants | Monogenic*: TNFAIP3* pathogenic variants |
| Autoantibodies | - | + | + |
| Other syndromes or diagnostic labels reported in patients with pathogenic variants in *RELA* or *TNFAIP3* (A20) | n/a | Sjogren syndrome  SLE (with nephritis)  ALPS  Neuromyelitis Optica  Shingles  Herpes | ALPS  Juvenile idiopathic arthritis  Rheumatoid arthritis  Sjogren’s syndrome  Psoriasis  Vitiligo  Type 1 Diabetes  Hypothyroidism  ITP  Pericarditis  Polyarteritis Nodosa  Nephrotic syndrome  PFAPA  Adult-Onset Stills Disease  Recurrent URTI  Immunoglobulin deficiency |

**Supplementary Table 3:** The table above compares the similarities and differences between classic polygenic BD, versus monogenic BD driven by pathogenic variants in *RELA* or *TNFAIP3* (A20). ‘+’ denotes presence of specific feature in some cases (not necessarily all cases).

Abbreviations: ALPS = Autoimmune Lymphoproliferative Syndrome; BD = Behcet’s Disease; HA20 = Haploinsufficiency of A20; ITP; immune thrombocytopenic purpura; PFAPA = Periodic Fevers, Aphthous stomatitis, Pharyngitis, Adenitis; RAID = *RELA*-Associated Inflammatory Disease; SLE = Systemic Lupus Erythematosus; URTI = upper respiratory tract infection
