# Supplementary material for: Case report: Novel variants in RELA associated with familial Behcet’s-like disease
Source: Front Immunol. 2023 Feb 28;14:1127085. doi: 10.3389/fimmu.2023.1127085 (PMC10011480; doi:10.3389/fimmu.2023.1127085)
Supplement: Supplementary file 4 [file DataSheet_1.docx]

**Methods NGS-Panel for RELA**:

A sequencing panel targeting 241 inflammatory and autoimmunity genes was designed and the Twist Biosciences Target Enrichment and Library Preparation Technology was used to generate NGS libraries. The captured sequences included all coding and untranslated exons with at least 10 bp of the flanking intronic sequence of all 241 genes. Libraries were sequenced on an Illumina MiSeq sequencer using the 150bp paired-end mode method. GATK best practice pipeline in combination with Gemini, Exomiser, and CNV-Atlas was applied to identify disease-associated single nucleotide variants (SNVs) and copy number variations (CNVs). Previously diagnosed patients and healthy parents and/or siblings served as positive and negative controls, respectively.

**List of sequenced genes below:**

| ACP5  ADA2  ADAM17  ADAR  AIM2  AIRE  AP1S3  BAG6  BIRC2  BIRC3  C1QA  C1QB  C1R  CARD14  CARD8  CASP10  CASP4  CASP5  CASP8  CD274  CD86  CEBPB  CFLAR  CGAS  CHUK  COPA  CTLA4  CXCL8  CYLD  DDX58  DNASE1  DNASE2  ECPAS  ELANE  ELMO1  FADD  GSDMD  HTATIP2  ICOS  IFIH1  IFNG  IKBKB  IKBKE  IKBKG  IL10  IL10RA | IL10RB IL12A  IL18RAP  IL1R1  IL1R2  IL1RAP  IL1RAPL2  IL1RN  IL2  IL36RN  IL6  IL6R  IRAK1  IRAK2  IRAK3  IRAK4  IRF3  IRF7  IRF8  IRF9  ISG15  JAK1  JAK2  JAK3  LACC1  LPIN2  LRBA  LRCH4  LYN  LYST  MALT1  MAP3K14  MAP4K4  MAPK12  MEFV  MLKL  MVK  MYD88  NCF2  NCF4  NCSTN  NFAT5  NFKB1  NFKB2  NFKBIA  NFKBIZ  NLRC4 | NLRP1  NLRP12  NLRP2  NLRP3  NLRP4  NLRP6  NLRP7  NLRP9  NOD2  NR2C2  OTUD7B  OTULIN  PAAF1  PDCD1  PDCD1LG2  PLCG1  PLCG2  POLA1  POMP  PRF1  PSMA1  PSMA2  PSMA3  PSMA4  PSMA5  PSMA6  PSMA7  PSMA8  PSMB1  PSMB10  PSMB11  PSMB2  PSMB3  PSMB4  PSMB5  PSMB6  PSMB7  PSMB8  PSMB9  PSMC1  PSMC2  PSMC3  PSMC4  PSMC5  PSMC6  PSMD1 | PSMD10  PSMD11  PSMD12  PSMD13  PSMD14  PSMD2  PSMD3  PSMD4  PSMD5  PSMD6  PSMD7  PSMD8  PSMD9  PSME1  PSME2  PSME3  PSME4  PSMF1  PSMG1  PSMG2  PSMG3  PSMG4  PSTPIP1  PSTPIP2  RBCK1  RC3H1  RC3H2  REL  RELA  RELB  RIPK1  RIPK2  RIPK3  RNASEH2A  RNASEH2B  RNASEH2C  RNF31  SAMHD1  SCAF11  SEM1  SH3BP2  SHARPIN  SKIV2L  SLC29A3  SPAG7  STAT1 | STAT2  STAT3  STAT4  STAT5A  STAT5B  STAT6  STXBP2  TAK1  TANK  TANK  TAX1BP1  TBK1  TBX21  TIRAP  TLR1  TLR2  TLR3  TLR4  TLR5  TLR6  TLR7  TMEM173  TNF  TNFAIP3  TNFRSF10C  TNFRSF10D  TNFRSF11A  TNFRSF1A  TNFRSF6B  TNIP1  TNIP2  TNIP3  TRADD  TRAF1  TRAF2  TRAF6  TREX1  TRNT1  TXNL1 | TYK2  UBE3C  UCHL5  UNC13D  USP14  USP18  USP43  WAS  WDR1  XIAP  ZBP1  ZC3H12A  ZFAND2A  ZFAND2B  ZFP36  ZFP36L1  ZFP36L2 |
| --- | --- | --- | --- | --- | --- |
